# Formation of Phases and Microstructures in Al-8Si Alloys with Different Mg Content

**DOI:** 10.3390/ma14040762

**Published:** 2021-02-06

**Authors:** Ni Tian, Guangdong Wang, Yiran Zhou, Chuncheng Liu, Kun Liu, Gang Zhao, Liang Zuo

**Affiliations:** 1 School of Materials Science & Engineering, Northeastern University, No. 3-11, Wenhua Road, Heping District, Shenyang 110819, China; w15041838726@163.com (G.W.); zhouyr@mail.neu.edu.cn (Y.Z.); zhaog@mail.neu.edu.cn (G.Z.); 2 Key Laboratory for Anisotropy and Texture of Materials (Ministry of Education), Northeastern University, No. 3-11, Wenhua Road, Heping District, Shenyang 110819, China; lzuo@mail.neu.edu.cn; 3 Engineering Training Center, Northeastern University, No. 3-11, Wenhua Road, Heping District, Shenyang 110819, China; liuccsy@sina.com; 4 Department of Applied Science, University of Quebec at Chicoutimi, 555, Boul. de L’University, Chicoutimi, QC G7H2B1, Canada; kun.liu@uqac.ca

**Keywords:** Al-8Si alloy, Mg content, Direct chill (DC) casting, thermodynamic calculation, solidification process

## Abstract

Mg-containing high-silicon aluminum alloy is a heat-treatable aluminum alloy that is now widely used in the aerospace and automotive industries because of its high specific strength, high wear resistance and corrosion resistance, low thermal expansion coefficient, and low cost. More attention has been paid to optimizing the microstructure to increase the performance of this type of aluminum alloy. In the present work, the solidification processes of Mg-free and Mg-containing (0.33–1.32%) Al-8Si alloys were analyzed by the experimental results combined with the thermodynamic calculation. The results showed that α-Al, Si, and Al_5_FeSi were in the Mg-free Al-8Si alloy ingots, while the Al_5_FeSi phases in alloys with Mg additions were transformed into π phases (Al_8_Mg_3_FeSi_6_) by the reaction L+Al_5_FeSi→α-Al+Si+Al_8_Mg_3_FeSi_6_. There was a binary eutectic reaction of L→α-Al+Al_5_FeSi when the Mg content exceeded 0.51% and the Fe content was higher than 0.17%. With the increase of Mg content, the volume of Mg_2_Si was gradually increased while the divorced eutectic phenomenon of the quaternary eutectic structure (α-Al+Si+Mg_2_Si+Al_8_Mg_3_FeSi_6_) was weakened and the eutectic structure was significantly refined.

## 1. Introduction

Al-Si alloy is widely used in the aerospace and automotive industries because of its high specific strength, wear resistance and corrosion resistance, low thermal expansion coefficient, and excellent castability that is incomparable to other aluminum alloys [1,2,3,4]. Many research results show that when a certain amount of Mg is added to Al-Si alloy, Mg_2_Si particles can be dissolved into the matrix by solid solution treatment and further precipitate as finer precipitates during aging to improve the mechanical properties of Al-Si alloy [5,6,7,8]. Zhu et al. [9] studied the effect of Mg on the microstructure of Al-2.4Si-(5.22–12.33)Mg (mass fraction, %, the same below) and found that the addition of Mg made the microstructure of the alloy more uniform. However, with the increase in Mg content, the eutectic Mg_2_Si changed from rod-like or lamellae to curved flakes with large eutectic spacing. Simultaneously, the addition of Mg resulted in a slight decrease in elongation of the alloy and a significant increase in strength. Huang et al. [10,11] studied the effect of Mg on the primary Mg_2_Si in Al-16Si-0.95Ni alloy and showed that the volume fraction of primary Mg_2_Si particles linearly increased with the increase in Mg content, but the average size of Mg_2_Si particles did not significantly change. When the Mg content was 3%, Mg_2_Si particles grew fastest along <100> of the Al matrix and formed octahedral shapes. However, primary Mg_2_Si particles changed from the octahedron shape into various complex structures with large sizes when further Mg was added (~10%). Alfonso et al. [12] studied the effect of Mg content on the microstructure of Al-6Si-3Cu-(0.59, 3.80, 6.78)Mg alloy and found that an increase in Mg content promoted the change of Cu-rich-phase Al_2_Cu into Q-phase Al_5_Cu_2_Mg_8_Si_6_, and the dissolution of the Q phase was more difficult than that of the Al_2_Cu phase. Li et al. [13] focused on Al-20%Mg_2_Si-3%Si alloy to study the microstructure and solidification process of hypereutectic Al-Mg-Si alloy. Primary Mg_2_Si phases were surrounded by the Al-Mg_2_Si binary eutectic and subsequently by the Al-Mg_2_Si-Si ternary eutectic structures. The primary Mg_2_Si phase at the centers showed dendritic or polyhedral morphologies. Al-Mg_2_Si binary eutectic cells with regular morphologies have flake-like Mg_2_Si surrounded by α-Al. Wu et al. [14] studied the effects of Cu on the microstructure and mechanical properties of Al-14.5Si-0.5Mg alloy, and it is reported that the Fe-containing phases in the alloy were β-Al_5_FeSi when the Cu content was 4.65% but were converted to Al_8_Mg_3_FeSi_6_ with a Cu content of 0.52%. In a study of Al-7Si-3Cu-(0–0.92)Mg alloy, Aguilera-Luna et al. [11,15] found the best modification effect of eutectic silicon particles and the smallest eutectic silicon size was achieved when the Mg content was 0.6% under the cooling rate of 4 °C/s. In conclusion, the reviewed research on the effect of Mg content on the microstructure of high-Si aluminum alloy is mainly focused on the primary Mg_2_Si phase with a relatively high Mg content. However, there are limited open studies on the effect of Mg content below 1.5% on the microstructure of high-Si aluminum alloy. In particular, there are few reports on the effect of Mg additions on the formation process and evolution mechanism of the second phase in Al-Si alloys. As a structural material, Al-Si alloys are required to have high corrosion resistance, high wear resistance, a low thermal expansion coefficient, and good strength, plasticity, and toughness. To improve the strength and plastic toughness of Al-Si alloy, the Mg content added to this type of alloy is generally no more than 1.5% to ensure that the solid solubility of Mg_2_Si in Al does not reach the upper limit of solubility, which is 1.85%, and avoid the appearance of a coarse primary Mg_2_Si phase.

There are many factors affecting the actual solidification process of aluminum alloy: not only alloy composition but also casting temperature, cooling rate, and casting pressure. Many studies have shown that the casting pressure has a significant effect on the morphology, microstructure, and second phases of aluminum alloy [16,17,18]. Therefore, the fixed direct chill casting (DCC) parameters, including the casting temperature, casting speed, cooling rate, and the casting pressure, were used to cast the experimental alloys in the current work to focus on the effect of Mg content on the type, morphology, size, and quantity of the second phases in Al-8Si alloy. The solidification process of the phases and microstructures in Al-8Si aluminum alloys with different Mg contents were analyzed in combination with the phase diagram and thermodynamic analysis to provide the experimental understanding on optimizing the composition and improving the overall performance of Mg-containing Al-Si alloy.

## 2. Materials and Methods 

The alloy ingots were prepared from commercial-purity Al (99.7%), electrolysis-grade copper, commercial-purity Mg, and Al-30Si master alloy. These raw materials were melted in an electrical resistance crucible furnace and direct chill (DC) cast into ingots (350 mm × 200 mm × 60 mm). All of the ingots were cast at 720 °C with a casting speed of 80 mm/min in air at atmospheric pressure. The actual cooling rate during the solidification in the present work was about 6–10 K/s from the center to the wall of ingot. The compositions of the alloy ingots in the present study are shown in Table 1.

Metallographic specimens with a size of 10 mm × 10 mm × 15 mm were cut from the cores of the six alloy ingots, and the observed surface was perpendicular to the direction with the maximum cooling gradient. The microstructure was examined with an Olympus GX71 optical microscope (OM) (Olympus Corporation, Tokyo, Japan) and a JSM 6510 scanning electron microscope (SEM) coupled with an INCAX-Sights energy dispersive spectrometer (EDS) (JEOL Ltd., Tokyo, Japan), and the EDS results are based on standardless quantitative analysis. Besides, the phase analysis was performed by an X′ Pert Pro MPD (Panalytical, Eindhoven, Holland) X-ray diffraction system with the test conditions of Cu Kα rays under the tube voltage of 40 kV and the tube current of 40 mA. The scanning rate was 2°/min with a step size of 0.02°. The XRD specimens in the present work were bulk samples by removing the surface stress, after etching with 5% NaOH aqueous solution for 3 minutes, on a grinded sample with #2000 grit wet sandpapers. In addition, the solidification simulation function in Pandat software (CompuTherm LLC, Middleton, WI, USA, 2016 version) was used to predict the non-equilibrium solidification processes of the experimental alloys. The Gulliver-Scheil model was used to simulate the non-equilibrium solidification calculation.

## 3. Results and Discussion

### 3.1. Effect of Mg Content on the Microstructure of DC-Casted Al-8Si Alloy

**Figure 1:**
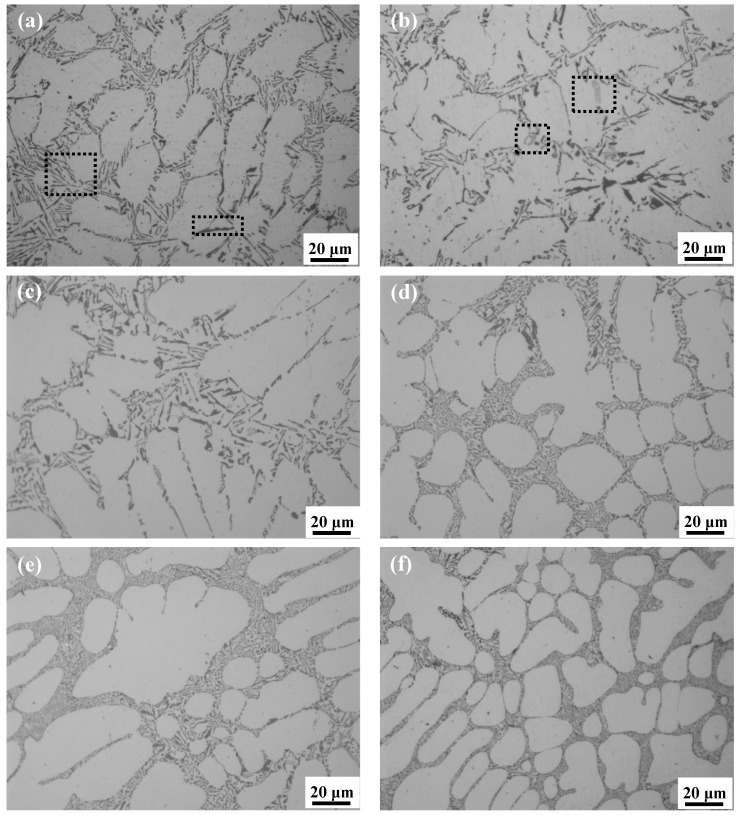
Optical microstructures of Al-8Si alloy ingots (**a**) without Mg and with (**b**) 0.33% Mg, (**c**) 0.51% Mg, (**d**) 0.78% Mg, (**e**) 0.99% Mg, and (**f**) 1.32% Mg.

The as-cast microstructures of DC-casted Al-8Si free of Mg and Al-8Si-(0.33–1.32)Mg alloy ingots are shown in Figure 1. There were many white α-Al dendrites and many needle and flake gray or dark-gray phases, which were all distributed along the grain boundary or interdendritically (as shown in Figure 1a, dashed rectangle area) in the Mg-free Al-8Si alloy ingot. With a small amount of Mg added (0.33% in Figure 1b and 0.51% in Figure 1c), the microstructure of the alloy ingots had no significant changes and there were still many needle and flake or block light-gray phases (as shown in Figure 1b dashed rectangle area). When the amount of Mg increased to 0.78% (Figure 1d), the eutectic structure, which was distributed along the grain boundary or interdendritically in the alloy ingots, was significantly refined. With further addition of Mg (0.99% in Figure 1e and 1.32% in Figure 1f), the eutectic structure of the alloy ingots became gradually refined.

### 3.2. Effect of Mg Content on the Phases of DC-Casted Al-8Si Alloy

**Figure 2:**
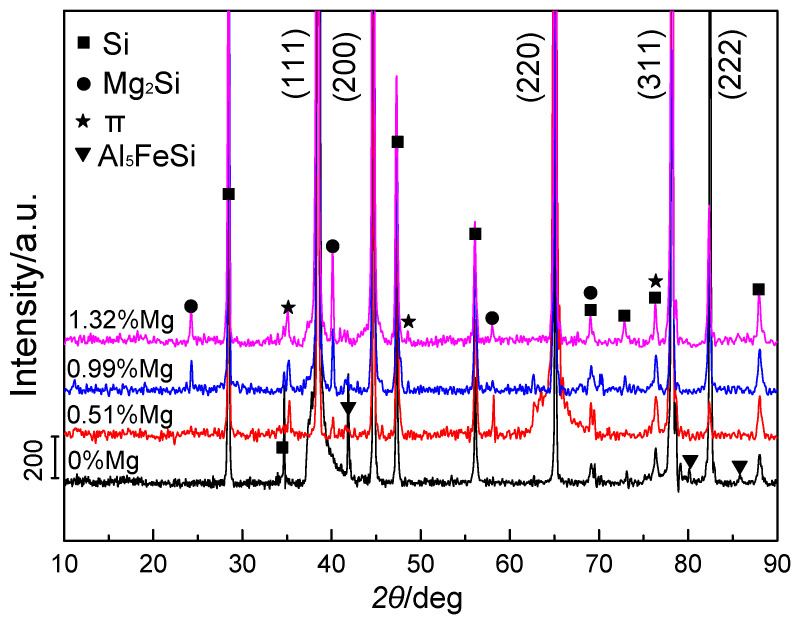
XRD patterns of Al-8Si alloy ingots without Mg and with 0.51%, 0.99%, and 1.32% Mg.

Figure 2 shows the XRD patterns of experimental alloys with various Mg additions. Three main types of alloy phases in Al-8Si alloy ingots without Mg were found: α-Al, Si, and Al_5_FeSi. When increasing the Mg additions, the intensity of the diffraction peak of Al_5_FeSi in Al-8Si-xMg alloy ingots weakened and almost disappeared at higher Mg contents (~1.32%). Moreover, additional diffraction peaks of the π phases (Al_8_Mg_3_FeSi_6_) and Mg_2_Si phases appeared in the alloy ingots and their intensity was slightly enhanced with increasing Mg contents. Therefore, it can be concluded from the XRD characterization that the predominant phase changes were from α-Al, Si, and Al_5_FeSi phases in Mg-free alloy to α-Al, Si, π (Al_8_Mg_3_FeSi_6_), and Mg_2_Si phases in Mg-containing Al-8Si-xMg alloy ingots.

**Figure 3:**
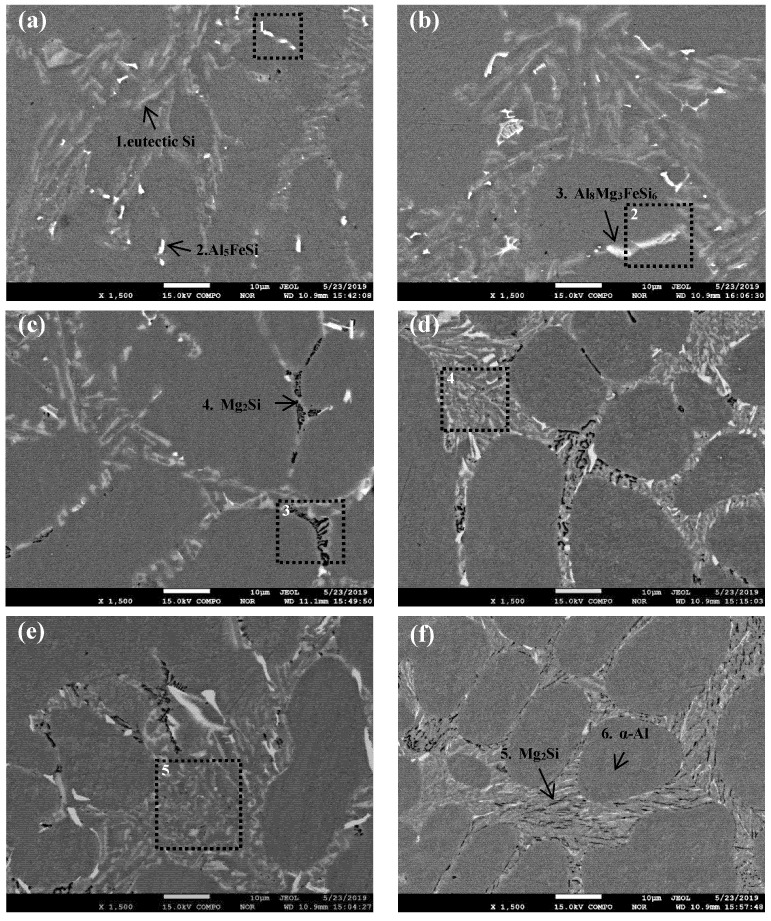
SEM images of Al-8Si alloy ingots (**a**) without Mg and with (**b**) 0.33% Mg, (**c**) 0.51% Mg, (**d**) 0.78% Mg, (**e**) 0.99% Mg, and (**f**) 1.32% Mg.

The SEM images of Al-8Si alloy and Al-8Si-(0.33–1.32)Mg alloy ingots are shown in Figure 3, and the typical EDS analysis of each alloy phase obtained from one large-enough detection point in Figure 3 are summarized in Table 2.

According to the XRD results in Figure 2 and Table 2, the gray needle-flake phases were eutectic Si phases (as shown in Figure 3a, Point 1), and the bright white needle or blocky phases were Al_5_FeSi phases (as shown in Figure 3a, Point 2). However, after a small amount of Mg was added, Al_5_FeSi phases were rarely observed and π phases (Al_8_Mg_3_FeSi_6_) appeared (Figure 3b, Point 3), indicating that Al_5_FeSi phases were transformed into π phases (Al_8_Mg_3_FeSi_6_) by the addition of Mg. Tebib et al., with Al-15Si-14Mg-4Cu alloy, Wu et al., with Al-14.5Si-4.5Cu-xMg alloy, Rincon et al., with A319 alloy, and Mbuya et al., with Al-Si alloy, have proven that the medium-rich Fe phases of Mg-containing Al-Si alloy are π phases (Al_8_Mg_3_FeSi_6_), which is consistent with the results of this study [19,20,21,22].

When the Mg content reached 0.51%, there were unevenly distributed black block or skeletal Mg_2_Si phases in the alloy (as shown in Figure 3c, Point 4). When the Mg content further increased to 0.78%, the eutectic reaction products (mainly eutectic Si phases) in the alloy were significantly refined, which was consistent with the observed metallographic results. Furthermore, the number of Mg_2_Si phases in the alloy gradually increased, and black-dotted Mg_2_Si phases appeared in the regions with eutectic reaction characteristics (as shown in Figure 3d, dotted area). Finally, when the Mg content was increased to 1.32%, the black Mg_2_Si phases in the alloy changed from medium black blocks or bone to smaller needle shapes with uniform distribution, and the number significantly increased. 

### 3.3. Effect of Mg Content on the Solidification Behavior of DC-Casted Al-8Si Alloy

**Figure 4:**
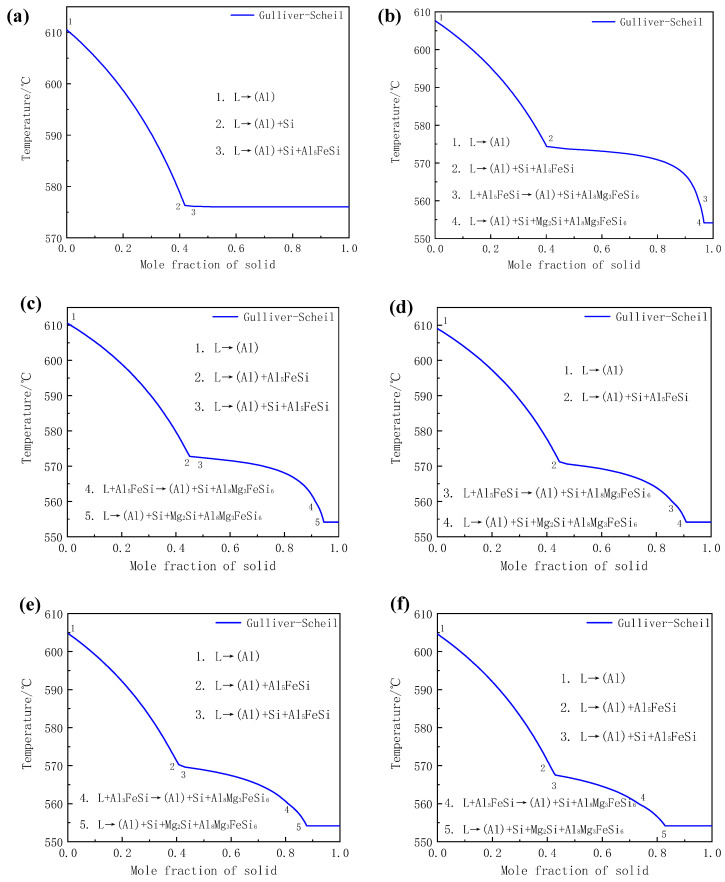
Calculated solidification curves of Al-8Si-xMg alloys under Gulliver-Scheil non-equilibrium conditions (**a**) without Mg and with (**b**) 0.33% Mg, (**c**) 0.51% Mg, (**d**) 0.78% Mg, (**e**) 0.99% Mg, and (**f**) 1.32% Mg.

Numerous studies indicate that the solidification reactions of various series of aluminum alloys, such as Al-Mg-Si, Al-Fe-Si, Al-Mn-Si, Al-Fe-Mn-Si, Al-Fe-Mn-Si, Al-Fe-Mn-Si, and Al-Cu-Fe-Mg-N aluminum alloys, can be well predicted by using the Gulliver-Scheil model, which were in good agreement with the actual experimental results [23,24,25]. Therefore, the Gulliver-Scheil model in Pandat software was performed in the present work to predict the solidification process of Al-8Si-(0–1.32)Mg-0.13Fe alloy, and the results are shown in Figure 4 and Table 3. It can be seen that Al, Si, and Al_5_FeSi phases formed during the solidification of Mg-free Al-8Si alloy. With the addition of Mg, additional Al_8_Mg_3_FeSi_6_ and Mg_2_Si besides Al, Si, and Al_5_FeSi were predicted and the types of alloy phases remained unchanged as the Mg content gradually increased to 1.32%, which was completely consistent with the XRD results in Figure 2 and the SEM observations in Figure 3. Therefore, based on the metallurgical microstructure analysis in Figure 3 and Backerud’s study [26] as well as the thermodynamic simulation with the Gulliver-Scheil model in Figure 4, the solidification reactions of experimental alloys can be summarized. In the Mg-free Al-8Si aluminum alloy (Figure 4a and Table 3), the α-Al dendrite, (α-Al+Si) binary eutectic, and (α-Al+Si+Al_5_FeSi) ternary eutectic were obtained by L→α-Al at 610.5 °C, L→α-Al+Si at 576.1 °C, and L→α-Al+Si+Al_5_FeSi at 576.0 °C, respectively. With the addition of 0.33% Mg (Figure 4b and Table 3), the L→α-Al+Si binary eutectic reaction disappeared during the solidification process, whereas L→α-Al, L→α-Al+Si+Al_5_FeSi, and L+Al_5_FeSi→α-Al+Si+Al_8_Mg_3_FeSi_6_ occurred successively at about 607.6 °C, 573.7 °C, and 559.6 °C, respectively. In other words, Al_5_FeSi was transformed into Al_8_Mg_3_FeSi_6_ with the addition of 0.33% Mg. There was a multiple eutectic reaction of L→α-Al+Si+Mg_2_Si+Al_8_Mg_3_FeSi_6_ when Al-8Si-0.33Mg aluminum alloy was cooled to 554 °C. However, no obvious quaternary eutectic structure (α-Al+Si+Mg_2_Si+Al_8_Mg_3_FeSi_6_) was observed in Al-8Si-0.33Mg alloy in Figure 3b, which could be due to the less-available Mg in liquid since Mg preferentially formed the Al_8_Mg_3_FeSi_6_ phase through the reaction of L+Al_5_FeSi→α-Al+Si+Al_8_Mg_3_FeSi_6_ at a higher temperature. When the Mg content in Al-8Si alloy increased to 0.51% (Figure 4c and Table 3), there was a binary eutectic reaction of L→α-Al+Al_5_FeSi that occurred at 574 °C. The other reactions did not change significantly except that the corresponding reaction temperature decreased slightly. It is worth noting that the L→α-Al+Al_5_FeSi binary eutectic reaction did not occur during the solidification of the alloy as the Mg content increased to 0.78% (Figure 4d and Table 3). As the content of Mg was further increased to 0.99% (Figure 4e and Table 3) and 1.32% (Figure 4f and Table 3), all of the solidification reactions were the same as those in Al-8Si-0.51Mg except that the reaction temperature corresponding to all reactions gradually decreased. In conclusion, the addition of Mg can transform Al_5_FeSi phase into Al_8_Mg_3_FeSi_6_. When the Mg content in Al-8Si alloy exceeds 0.51%, the binary eutectic reaction L→α-Al+Al_5_FeSi occurs during the solidification process at the current Fe level (~0.2%). In addition, with the increase in Mg content, the reaction temperature during the solidification gradually decreases.

Most notably, it can be observed from Figure 3 that there were significant differences in the morphology of phases with different Mg content. The principal reason for this is that the percentage of each alloy phase in the polyphase eutectic structure formed during the solidification was significantly different. For instance, the (α-Al+Si+Al_5_FeSi) ternary eutectic structure only had the two-phase typical laminar structure characteristics of the alternating nucleation of α-Al and Si, because there were few Al_5_FeSi phases in the Mg-free Al-8Si alloy due to the Fe content in the alloy being less than 0.2%. During the reaction of L→α-Al+Si+Al_5_FeSi at 576.0 °C in the Mg-free Al-8Si alloy, the heterogeneous nucleation of α-Al and Si phases easily occurred on the surface of the pre-formed α-Al and Si at a higher temperature (Reaction 2 in Figure 4a), while a very small amount of blocky Al_5_FeSi phase slowly formed at the grain boundaries and the interdendrites during the solidification. Therefore, Al_5_FeSi presented as divorced eutectic morphology, as indicated in Region 1 in Figure 3a. Similar divorced eutectic microstructures were also observed in Mg-containing alloys, such as Al_8_Mg_3_FeSi_6_ in the alloy with 0.33 Mg (Region 2 in Figure 3b) and Mg_2_Si in the alloy with 0.51 Mg (Region 3 in Figure 3c). The Mg_2_Si phase in (α-Al+Si+Mg_2_Si+Al_8_Mg_3_FeSi_6_) quaternary eutectic was gradually refined and showed typical lamellar eutectic characteristics as the Mg_2_Si content increased with the increase of Mg content (Region 4 in Figure 3d and Region 5 in Figure 3e). Two things lie behind this quaternary eutectic refinement and more eutectic characteristics with the increase in Mg content. One is that the solidification temperatures of all kinds of alloy phases in the Al-8Si-(0.33–1.32)Mg alloy gradually decreased as the Mg increased. The other is that the supercooling of the front edge of the solid-liquid interface increased during the actual solidification of Al-8Si-(0.33–1.32)Mg due to the addition of Mg.

## 4. Conclusions

(1) There were α-Al, Si, and Al_5_FeSi in the Mg-free Al-8Si alloy, while the Al_5_FeSi phases were transformed into π phases (Al_8_Mg_3_FeSi_6_) by the reaction L+Al_5_FeSi→α-Al+Si+Al_8_Mg_3_FeSi_6_ with the addition of Mg.
(2) A binary eutectic reaction L→α-Al+Al_5_FeSi was observed when the Mg content exceeded 0.51% and the Fe content was greater than 0.17%.
(3) With increasing Mg content, the solidification reactions remained unchanged. The number of Mg_2_Si was gradually increased while the divorced eutectic phenomenon of quaternary eutectic structure gradually weakened and the quaternary eutectic structure was significantly refined.

## Figures and Tables

**Table 1:** Compositions of the alloy ingots in the present study (mass %).

No.	Mg	Si	Fe	Al
1	-	7.83	0.18	Bal.
2	0.33	8.07	0.18	Bal.
3	0.51	7.53	0.19	Bal.
4	0.78	7.61	0.16	Bal.
5	0.99	8.11	0.20	Bal.
6	1.32	7.96	0.17	Bal.

**Table 2:** EDS results of the points in Figure 3. (Atomic fraction %).

Points	Al	Si	Fe	Mg
1	90.82	9.18	-	-
2	84.48	6.89	8.63	-
3	74.12	15.85	3.10	6.94
4	81.36	9.24	-	9.40
5	76.46	15.64	-	7.90
6	98.16	1.48	0.06	0.30

**Table 3:** Main reactions observed from the thermal analysis diagrams of the experimental alloys.

Alloy Code	Reaction Number	Temperature (°C)	Type of Reaction
1 (0 Mg)	1	610.5	L→α-Al
	2	576.1	L→α-Al+Si
	3	576.0	L→α-Al+Si+Al_5_FeSi
2 (0.33% Mg)	1	607.6	L→α-Al
	2	573.7	L→α-Al+Si+Al_5_FeSi
	3	559.6	L+Al_5_FeSi→α-Al+Si+Al_8_Mg_3_FeSi_6_
	4	554.2	L→α-Al+Si+Mg_2_Si+Al_8_Mg_3_FeSi_6_
3 (0.51% Mg)	1	610.5	L→α-Al
	2	574.1	L→α-Al+Al_5_FeSi
	3	572.1	L→α-Al+Si+Al_5_FeSi
	4	559.3	L+Al_5_FeSi→α-Al+Si+Al_8_Mg_3_FeSi_6_
	5	554.2	L→α-Al+Si+Mg_2_Si+Al_8_Mg_3_FeSi_6_
4 (0.78% Mg)	1	609.0	L→α-Al
	2	570.6	L→α-Al+Si+Al_5_FeSi
	3	559.8	L+Al_5_FeSi→α-Al+Si+Al_8_Mg_3_FeSi_6_
	4	554.2	L→α-Al+Si+Mg_2_Si+Al_8_Mg_3_FeSi_6_
5 (0.99% Mg)	1	604.8	L→α-Al
	2	572.8	L→α-Al+Al_5_FeSi
	3	569.6	L→α-Al+Si+Al_5_FeSi
	4	559.4	L+Al_5_FeSi→α-Al+Si+Al_8_Mg_3_FeSi_6_
	5	554.2	L→α-Al+Si+Mg_2_Si+Al_8_Mg_3_FeSi_6_
6 (1.32% Mg)	1	604.6	L→α-Al
	2	570.1	L→α-Al+Al_5_FeSi
	3	566.9	L→α-Al+Si+Al_5_FeSi
	4	559.2	L+Al_5_FeSi→α-Al+Si+Al_8_Mg_3_FeSi_6_
	5	554.2	L→α-Al+Si+Mg_2_Si+Al_8_Mg_3_FeSi_6_

## Data Availability

The data presented in this study are available on request from the corresponding author.
